# An Investigation of Influencing Factors on Practical Sub-Diffraction-Limit Focusing of Planar Super-Oscillation Lenses

**DOI:** 10.3390/nano8040185

**Published:** 2018-03-22

**Authors:** Yiting Yu, Wenli Li, Haoyong Li, Muyuan Li, Weizheng Yuan

**Affiliations:** 1 Key Laboratory of Micro/Nano Systems for Aerospace, Ministry of Education, Xi’an 710072, China; Wenlili_nwpu@163.com (W.L.); lyhaoyong@163.com (H.L.); lixiaomu2110605@163.com (M.L.); yuanwz@nwpu.edu.cn (W.Y.); 2 Key Laboratory of Micro- and Nano-Electro-Mechanical Systems of Shaanxi Province, Northwestern Polytechnical University, Xi’an 710072, China

**Keywords:** super-oscillation lenses, focusing, sub-diffraction-limit, influencing factors

## Abstract

Planar super-oscillation lenses (SOLs) can fulfill super-resolution focusing and nanoscopic imaging in the far field without the contribution of evanescent waves. Nevertheless, the existing deviations between the design and experimental results have been seldomly investigated, leaving the practical applications of SOLs unpredictable and uncontrollable. In this paper, some application-oriented issues are taken into consideration, such as the inevitable fabrication errors and the size effect of the designed SOLs, with the aim of providing an engineering reference to elaborately customize the demanded focusing light field. It turned out that a thicker structural film makes the focal spots enlarged, while the sloped sidewalls just weaken the intensity of the focal hotspot. Furthermore, the focal lengths are diminished with the decrease of device size, while the focal spots are enlarged. This research will promote the wide-spread applications of SOLs for sub-diffraction-limit far-field focusing in the areas of nanoscopy and high-density optical storage.

## 1. Introduction

Optical lenses play an absolutely indispensable role in the optics-related industry, in which their focusing properties are of vital importance for the imaging quality and accuracy of the post-processing data. The resolving power of traditional optical lenses is subjected to the physical limit due to the loss of the spatial high-frequency Fourier components in the far field, which is the famous Rayleigh diffraction limit, defined as 0.61λ/NA (λ being the working wavelength, and NA being the numerical aperture of the lens) [1]. To solve this problem, scientists have made great efforts in the past decades. They have tried to take advantage of the manipulation of the surface plasmons of evanescent waves to realize super-resolution in the near field, such as the near-field scanning optical microscope [2] (NSOM), superlens [3], and plasmonic lens [4,5], mainly for the applications of super-resolution imaging [6,7] and nanolithography [8,9]. However, the evanescent waves attenuate exponentially due to the energy loss in the medium and cannot propagate to the far field or even the quasi-far field, imposing a fatal restriction for practical purposes. Our recent research on utilizing the polarization state of incident light to achieve the superfocusing capability in the quasi-far field may provide a feasible solution [10,11]. On the other hand, the optical super-oscillation phenomenon is the delicate interference of the far-field propagating waves, and it can be engineered to achieve a sub-diffarction-limit-focusing hotspot without the contribution of evanescent waves [12,13]. Besides, the experimental results are obtained in the far field from the output surface of super-oscillation lenses (SOLs) [14,15,16]. Currently, SOLs have found various uses in super-resolution imaging [17,18,19], high-density optical storage [20], and biomedicine [21]. 

**Figure 1:**
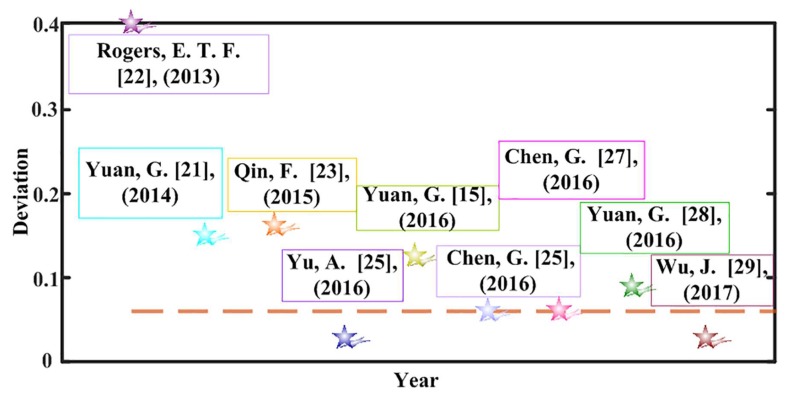
The deviation of the focusing properties of SOLs in recent publications.

Note that the high-quality fabrication of nanoscale devices still remains a worldwide challenge, and the fabrication imperfections more or less work on the performance of the micro/nano devices. Furthermore, it has been a common phenomenon that the experimental results do not always come out the same as the theoretical ones; we just summarize the deviation between the theoretical results and the measured ones from the recent publications [22,23,24,25,26,27,28,29], as shown in Figure 1. What we can clearly observe in Figure 1 is that most of the deviation is above 6%, while the relatively high deviation may impose a certain restriction on the practical application of the devices. Meanwhile, the literature on the fabrication imperfections generated from focused ion beam (FIB) milling such as the slot width error [30], the surface roughness error [9,31], and the sloped sidewalls [32,33,34], will be discussed. To provide some valuable practical advice for the more precise customization of the required light contours, three planar SOLs with distinct focal lengths are designed and fabricated to investigate the far-field focusing properties. It is noteworthy that we employ a multi-objective and multi-constraint optimization model to fulfill the sub-diffraction-limit light patterns through the vectorial angular spectrum (VAS) theory. To rapidly acquire a precise control over the light field far away from the lens surface, the fast Hankel transformation is also applied, and the method has been reported in our previous research [35,36]. As a consequence, both the simulated and measured focal sizes, along with the theoretical ones, all beat the calculated Rayleigh diffraction limit. More significantly, from the perspective of practical application, we mainly place an emphasis on the application-oriented issues such as the fabrication imperfections and size effect. To investigate the detailed error-tolerances generated from the fabrication imperfections of the SOLs, the specific error sources such as the structual layer thickness and the sloped sidewalls are investigated, and the fabrication errors are all characterized through the Atomic Force Microscopy (AFM). Furthermore, the corresponding simulations are finished to acquire a quantitative analysis of the fabrication imperfections. The results tell us that the slot width and surface roughness exhibit little influence on the focusing performance, but the film thickness and sloped sidewalls seem to influence greatly the focusing properties of SOLs. Moreover, through exploring the size effect, we find out that both the simulated and measured focal lengths are diminished with the decrease of device size, while the full-width at half-maximum (FWHM) of the foci is enlarged. The conclusions are in accordance with our previous studies [37,38]. This research will provide an indispensable technological reference for the design of the planar SOLs, which may find their promising applications during the integration into compact and cost-effective optical systems.

## 2. Design and Experimental Preparations

**Figure 2:**
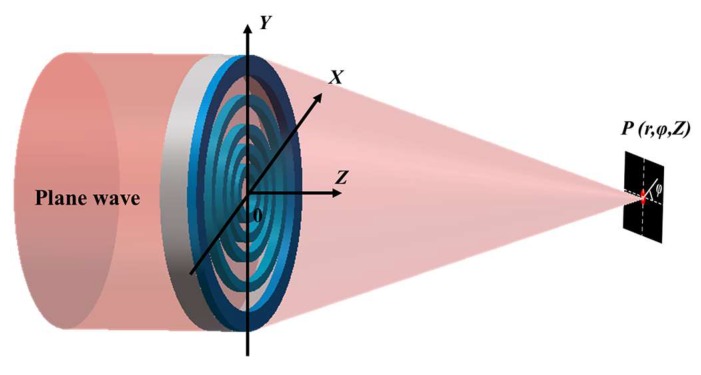
Schematic illustration of sub-diffraction-limit focusing by a planar super-oscillatory lens (SOLs).

The focusing behavior of planar SOLs can be ascribed to the delicate interference of diffracted beams in the light field by the specifically designed structural mask, and the schematic of sub-diffraction-limit focusing can be seen in Figure 2. The specific optimization process has been implemented through a multi-objective and multi-constraint genetic algorithm (GA) based on the VAS theory. The fast Hankel transformation is applied to accelerate the computational processes, and the whole algorithm has been elaboratedly depicted in our recent research [35,36]. 

For clarity, three SOLs with various focal lengths, i.e., *f*_1_ = 4 μm, *f*_2_ = 7 μm, and *f*_3_ = 10 μm, are denoted as the sample #1, #2, #3 in the following discussion, respectively. All the SOLs immersed in the oil (*n* = 1.515) are illuminated by the 640 nm laser source. The maximum radius of the mask is set to be 10 μm, with the total ring number of 100, making a minimum feature size (ring width) of 100 nm. The detailed design parameters and the corresponding calculated Raleigh diffraction limit for SOLs #1~#3 are given in Table 1, from which we can see that with the increase of focal length, the corresponding imaging quality is degraded, and the resolving power is reduced. According to the proposed optimization procedure, the transmittance distributions of the amplitude-type masks are achieved according to the different requirements, which are also listed in Table 1. For the binary amplitude annular mask, the contained concentric rings are initially set to be equidistant, and each ring can have either unit or zero transmittance, so the binary amplitude transmittance is encoded straightforward using the two digits {0, 1}. To describe the SOLs (which might contain several hundred rings) more compactly, the transmittance value *t_i_* is encoded from the first ring (innermost) to the *N*th ring (outermost) by continuously transforming every fourth successive binary digit into one hexadecimal digit. Take the sample SOL #2, for instance; the first hexadecimal digit “A” denotes the real transmittance values of “1010”.

**Figure 3:**
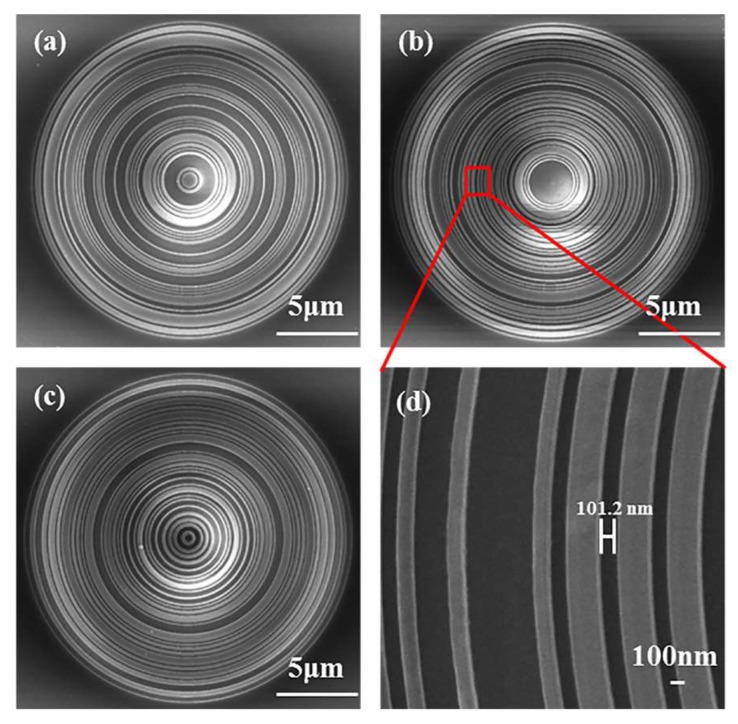
SEM diagrams of (**a**) SOL #1; (**b**) SOL #2; (**c**) SOL #3; and (**d**) the enlarged view of SOL #2.

In this study, all the samples are fabricated by the FIB milling, and the amplitude-type structures are characterized with an 80-nm-thick aluminium film deposited on the glass substrate via the electron-beam evaporation. A 10-nm-thick chromium layer is deposited on the substrate to enhance the adhesion between the glass substrate and the aluminium film. Figure 3 shows the scanning electron microscopy (SEM) images for the three samples and the enlarged view of sample #2, seeing the minimum annular radius is 101.2 nm. In view of the inherent problems brought by the fabrication techniques such as the dimensional deviation, the surface roughness, the sloped sidewalls seen from the SEM images, the influence of these imperfections, and the size effect on the focusing properties of SOLs will be further discussed in the following.

## 3. Results and Discussions

### 3.1. Characterization of Focusing Properties

To explore the far-field electromagnetic focusing characteristics of the designed planar SOLs, an analytical model is established firstly, as previously depicted in Figure 2. The simulation model is physically solved through a rigorous full-wave three-dimensional (3D) finite-difference time-domain (FDTD) electromagnetic method with the metallic-film-coated nanostructured mask immersed in the oil medium. The whole device is normally illuminated in the *Z* axis by a linearly polarized plane wave along the *X* axis of 640 nm wavelength. During the process of simulation, the anti-symmetric and symmetric boundary conditions are applied in the *X* axis and *Y* axis to make the whole computation storage drop sharply. The mesh sizes for *X* axis, *Y* axis, and the optical axis (*Z* axis) are all set to 20 nm. The values of permittivity (ε) and permeability (μ) for the metallic material are taken from Ref. [39].

**Figure 4:**
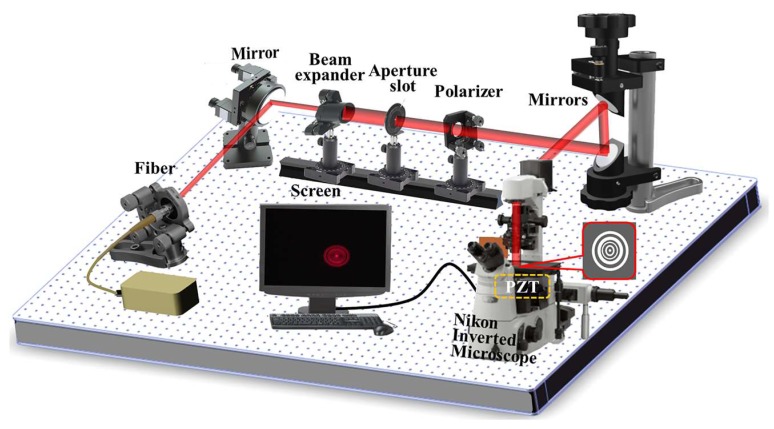
Schematic of optical measurement setup for SOLs’ electromagnetic focusing properties. CCD: Charge Coupled Device. PZT: Piezoelectric Transducer.

**Figure 5:**
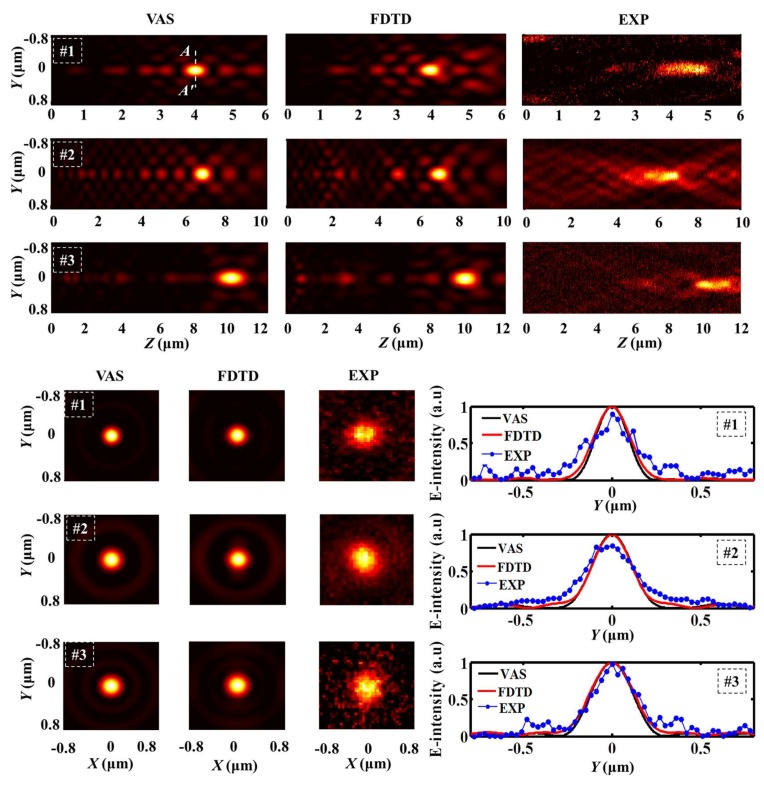
Top: electric intensity distributions in *Y*-*Z* planes of the three samples through VAS, FDTD simulation, and experiment. Bottom: Intensity profiles in the focal planes of the three samples obtained from VAS, FDTD simulation, and experiment. Besides, the corresponding normalized intensity distributions in the focal planes of the three samples are also shown.

During the process of practical measurement, the desired sub-diffraction-limit focusing hotspots are captured through the optical setup as shown in Figure 4. The transmitted light patterns through the samples are collected by a 100×/1.4 Nikon inverted oil immersion microscope objective (Nikon, Tokyo, Japan). Additionally, the image contours are recorded by a CCD camera (Nikon, Tokyo, Japan, the minimum image size is 0.3 μm) and scanned in Z axis with the step of 0.02 μm driven by the E-816 piezo controller (Physik Instrument, PI, Karlsruhe, Germany). In Figure 5, the simulated and measured light contours in the *Y*-*Z* planes are presented compared with the theoretical light intensity profiles. The normalized intensity distributions in the transverse focal plane along the dashed line *A*-*A* passing through the focal points in the three cases are also demonstrated in Figure 5, which shows that the simulated focal planes agree much better with the VAS calculations, while some discrepancies can be seen in the measured results. Note that the measured intensity of side lobes in the focal planes seems to be much larger than both the VAS calculated results and simulated contours, which can be attributed to the background noise of the circumstances, making the focal details blurred. To quantitatively analyze the hotspots in the focal plane, the VAS calculation and simulated normalized intensity of the FWHMs are numerically derived to compare with the measured results. Furthermore, the theoretical, simulated, and experimental focal lengths and FWHMs are all shown in Table 2, respectively. The non-conformity of the focal lengths between the simulated and the experimental ones might be accounted for by the imprecise estimation of the structural surface during the process of experiment. Although the side lobes of the experimental testing look much more severe than the VAS calculation and FDTD simulation, the focusing FWHMs acquired from the three samples are all beyond the calculated Rayleigh diffraction limit in the focal planes.

The above simulation and experiment are carried out at the wavelength of 640 nm without considering the chromatic effect. Chromatism may cause serious performance degradation of the optical imaging system, especially for the diffraction imaging. For practical application, the dispersion features of the diffractive devices cannot be ignored. The focusing property of the SOLs sample #1, #2, and #3 at other two wavelengths, λ = 532 nm and 730 nm, is examined. To quantitatively analyze the dispersion features of the SOLs, the experimental focal lengths and FWHMs are presented in Table 3. Obviously, the focal lengths are shortened as the wavelength increases, while the focal spots are enlarged, making the imaging quality degraded.

### 3.2. Fabrication Imperfections

**Figure 6:**
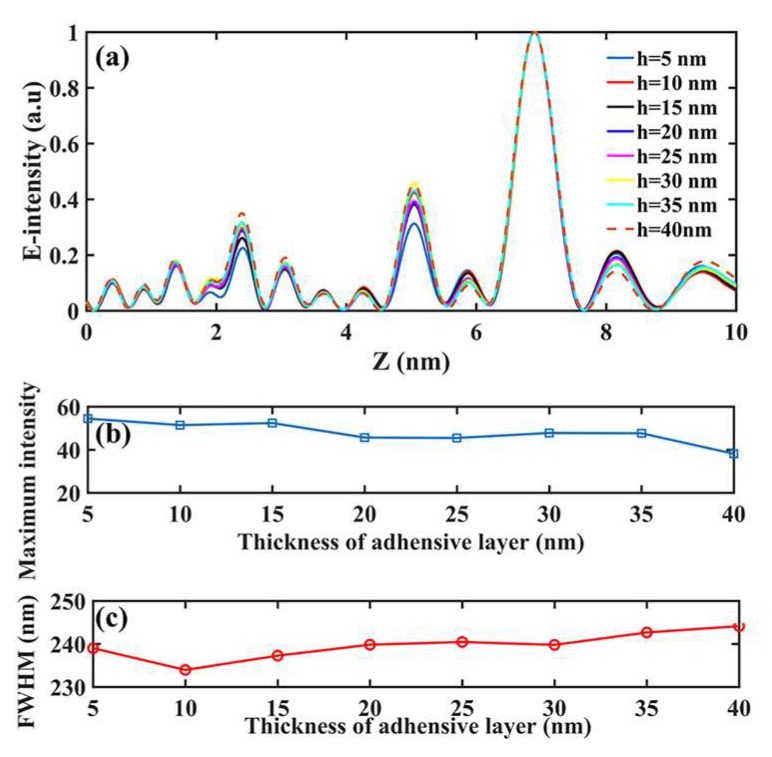
The changing rules of (**a**) the focusing properties, (**b**) the maximum intensity of the main hotspot, and (**c**) the FWHM with the increased thickness of adhesive layer.

Actually, there indeed exist some distinctions between the theoretical results and experimental ones as the Figure 1 shows above, in which the fabrication errors may play a non-ignorable role. To investigate the error-tolerance of the focusing properties of planar SOLs, we paid much attention to the common fabrication imperfections of FIB milling such as the slot width, surface roughness, structural layer thickness, and the sloped sidewalls. Although the chromium layer is just applied to enhance the adhesive force between the glass substrate and the aluminum film, its thickness should also be examined to see whether it affects the focusing properties of SOLs. According to our process capability, the thickness deviation of the customized metal film can be controlled within ±5 nm by employing electron-beam evaporation. To acquire much more complete influencing rules about the film thickness, we changed it from 5 nm to 40 nm with a 5-nm interval to investigate the variation of the focusing properties of SOLs in the simulation. For clarity, we just took the sample whose focal length is 7 μm as an example in the following discussion. The corresponding results can be seen in Figure 6, in which the axial intensity distribution shows no big difference with the increased thickness of adhesive layer. Furthermore, the maximum intensity of the focal spot gradually decreases as the adhesive layer becomes thicker due to the growing of propagation loss in the metal. However, the FWHMs are gradually enlarged when the adhesive layer is increased.

**Figure 7:**
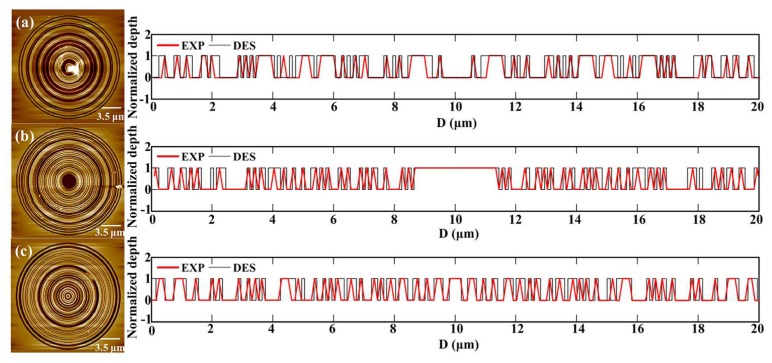
The fitted AFM cross-sectional diagram of the three SOLs with different focal lengths: (**a**) *f* = 4 μm, (**b**) *f* = 7 μm, (**c**) *f* = 10 μm.

**Figure 8:**
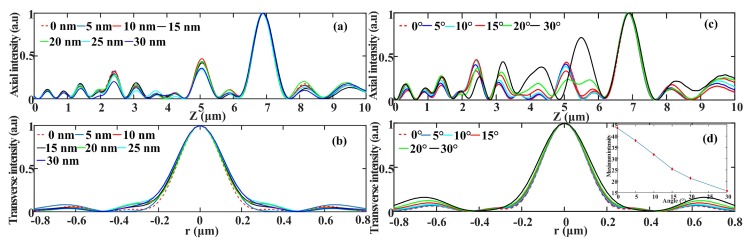
(**a**,**b**) The focusing performance of slot width deviation changing from 0 nm to 30 nm; (**c**,**d**) The focusing performance with the inclined sidewalls changing from 0 to 30°.

Figure 7 gives the AFM diagrams for the three SOLs with different focal lengths, and the corresponding slot width error is shown as well. It should be pointed out that we obtained the AFM diagrams via the curve fitting method, in case the probe did not reach the bottom of the grooves during the measurement. From the cross-sectional view of the as-fabricated SOLs’ geometrical profiles, we can obviously see that the FIB milling encountered a severe problem showing sloped sidewalls [32,33,34], attributed to its intrinsic physically sputtering etching mechanism. This issue has been neglected before by many researchers and has a close relationship with the aspect ratio (depth to width) of the etched nanostructures. In order to verify the error-tolerance of the slot width for the planar SOLs, we just set the deviation of each ring from 5 nm to 30 nm with a 5 nm interval. The simulated results are shown in Figure 8a,b; we can demonstrate that with the increase of slot width, the focal lengths stay almost the same, while the FWHMs are slightly enlarged. Figure 7 shows that the FIB milling will make obvious sloped sidewalls of the structure. By setting the sloped angle from 0 to 30°, the simulated focusing characteristics are presented in Figure 8c,d, from which we can see that with the increase of the sloped angle, the focal lengths almost keep constant while the FWHMs are enlarged, deteriorating the imaging quality of SOLs. However, the maximum intensity of the main focal spot weakens gradually as the sloped angle increases, also making the practical focusing performance degraded to some extent. It should be noted that the sloped sidewalls in the practical FIB milling will not be as large as 30°; we just try to declare an obvious influencing rule. 

**Figure 9:**
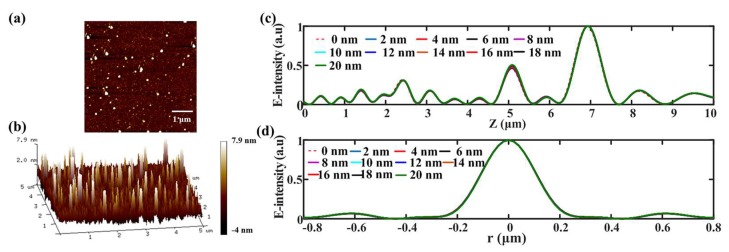
(**a**,**b**) The surface roughness characterized by AFM; The axial intensity distribution (**c**) and the transverse intensity distribution (**d**) of SOLs with the increase of surface roughness.

A smooth metallic film can always help to reduce the surface scattering loss during the focusing process, but the surface quality of the deposited metallic film does not always behave smoothly [9,31]. Taking this into account, we characterize the surface topography of the aluminum film utilizing the AFM. The corresponding measured results are displayed in Figure 9a,b. What we can infer from Figure 9 is that the root mean square (RMS) for surface roughness of the film is around 2 nm over a 5 × 5 μm^2^ region. To make a survey of the error-tolerance of the surface roughness, the RMS is changed from 0 to 20 nm with a 2-nm interval. The simulated results can be found in Figure 9c,d, from which we can see that the focusing properties of SOLs almost keep unchanged as the roughness increases.

**Figure 10:**
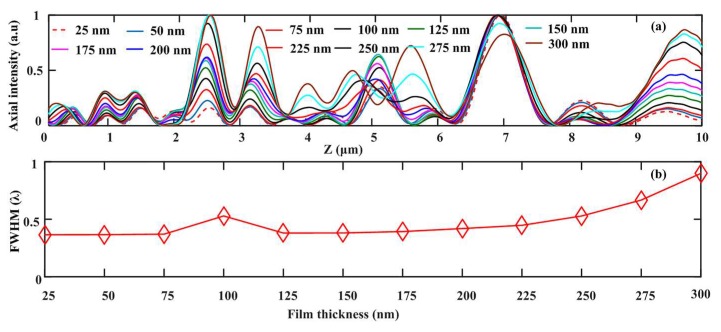
(**a**) The axial light intensity curve and (**b**) the changing FWHMs of the main foci as the thickness of structural film grows.

Taking account of these kinds of amplitude-type annular nanostructures, the thickness of the structural film may impose a significant role on the focusing characteristics of SOLs. In this situation, we change the thickness of the aluminum film from 25 nm to 300 nm with a 25-nm interval to obtain the light distribution along the optical axis, which can be seen in Figure 10a. Figure 10a shows that the designed main foci keep unchanged, while the intensity of the secondary foci are enhanced as the film thickness increases, weakening the imaging property of SOLs. Figure 10b clearly illustrates that the focal spots are enlarged with the increasing thickness. As a result, the optimal thickness is suggested to be 80 nm considering the skin depth of the structural material.

### 3.3. Size Effect

**Figure 11:**
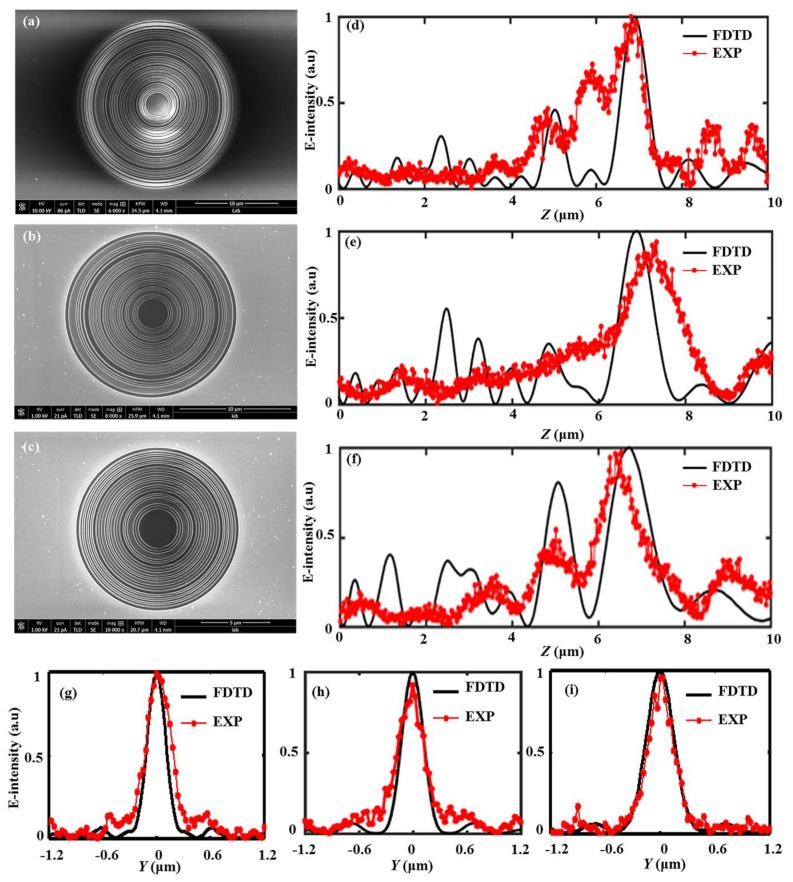
SEM images of the three samples ((**a**) *R* = 10 μm, (**b**) *R* = 8 μm, (**c**) *R* = 6 μm) and the corresponding axial intensity distribution along the optical axis for (**d**) #2-1, (**e**) #2-2, and (**f**) #2-3; Bottom: The normalized intensity line of the hotspots with their FWHMs for (**g**) #2-1, (**h**) #2-2, and (**i**) #2-3.

The plasmonic lens formed by 2D nanometric cross-shaped aperture arrays for Fresnel-region focusing was investigated by truncating the size of the arrays to vary the NA of the lens [40]. In addition, we also investigated the effect of lens size on the focusing performance of plasmonic lenses before and concluded that a larger lens size makes for better focusing behavior as a design [37]. To investigate the size effect on the focusing characteristics of SOLs, the radius of the sample #2 is gradually truncated from 10 μm to 6 μm at a 2-μm interval and the corresponding truncated cases are labeled as #2-1, #2-2, #2-3, respectively. Figure 11a–c displays the SEM diagrams of the three samples for different radii. Meanwhile, the axial light intensity distributions for the three samples are demonstrated in Figure 11d–f, and some differences can be seen between the simulated results and the experimental ones, which can be attributed to the approximate determination of the structural surface during the optical characterization. In addition, the transverse focusing properties are investigated by calculating the simulated and experimental normalized intensity distributions in the focal planes, which are shown in Figure 11g–i. The derived focal lengths and FHWMs are listed in Table 4, from which we can acquire that as the size decreases for SOL #2, focal shift phenomenon happens. It turns out that the FWHMs in the focal plane are gradually enlarged with the decrease of the device size, and this may be due to the enhanced diffraction effect [37,38].

Admittedly, the practical structural layer thickness and the specific feature size are not taken into account in the design process, bringing out the fact that the theoretical results are much smaller than those of the FDTD simulation. Figure 11 clearly depicts the size effect of SOLs, and we can clearly observe that the focal spots are enlarged, while the focal lengths are reduced as the radius of SOLs decreases. Since the fabrication imperfections cannot be avoided in the practical sample preparation, we can only make some adjustments to reduce the influence of the fabrication imperfections at the greatest extent. To make the FIB milling results more acceptable, the metal film with a smaller grain size should be used to form the structural layer. The other fabrication imperfections such as the sloped sidewalls, surface roughness, and slot width deviation can be controlled by optimizing the FIB milling process parameters, e.g., energy and diameter of the ion beam. As the device size decreases, namely, the number of transparent rings decreases correspondingly, the manipulation of light field by SOLs is not the same as before. According to the Fourier optics theory in the frequency domain, the sub-diffraction-limit light contours in the focal plane could be explained by the destructive annular-interference of the Fourier frequency components, which can help to bring some valuable information beyond the cut-off frequency caused by the finite aperture size of the lens based on the super-oscillation phenomenon.

## 4. Conclusions and Outlook

In summary, to give some valuable suggestions for the more precise acquisition of the desired focusing properties of planar SOLs, we implement some application-oriented investigations such as the fabrication imperfections and the size effect. Firstly, three SOLs with various focal lengths are designed, and the far-field electromagnetic focusing characteristics are explored experimentally. Both the theoretical and measured results are compared with those obtained from the 3D FDTD simulations; as imagined, all the results beat the Raleigh diffraction limit. Since there always remain some discrepancies between the theoretical results and experimental ones, we investigate the influences of the fabrication imperfections such as the slot width, surface roughness, structural layer thickness, and sloped sidewalls on the focusing properties of the planar SOLs. The results show that a thicker structural layer makes the focal spot enlarged, and the surface roughness seems to impose little influence on the focusing properties of SOLs. Besides, the slot width tolerance can be set less than ±15 nm to keep the focusing properties unchanged, while the sloped sidewalls weaken the intensity of the focal spot, leading to a poor imaging performance. Considering the practical applications, the size effect is also examined. It turns out that the focal lengths are diminished with the decrease of device size, while the FWHMs are enlarged. Note that chromatism may cause serious performance degradation of the optical imaging system, especially for diffraction imaging. Consequently, the operating bandwidth of the SOLs should be expanded to a large extent to satisfy a much wider application need, and the roadmap to realize an achromatic lens for these kinds of nanostructures can be realized through the optimization method [14,28,41]. The study provides a practical reference for the precise customization of the required light patterns of planar SOLs. A further study considering the design of achromatic SOLs will be also performed. Faced with the imperative application requirements in the complex optical system, the transformation from a single lens to a hybrid lens becomes an inevitable trend [12,42]. Furthermore, the manufacturing robustness and morphology errors, as well as the efficiency optimization, are all noteworthy in the future research.

## Figures and Tables

**Table 1:** Design parameters and transmittance functions of the optimized binary amplitude-type masks.

SOL	*R* (μm)	*f* (μm)	Calculated Rayleigh Diffraction Limit	Transmittance Function *t_i_*
#1	10	4	0.44λ	04FF2 90754 FDFA2 FD503 B198C
#2	10	7	0.49λ	FFFD 41351 22B24 BDA80 E89663
#3	10	10	0.57λ	CE4CE EB177 5217C 14904 8478E

**Table 2:** The VAS, simulated, and experimental focal lengths, and FWHMs, for the three samples.

SOL	*f* (μm)			FWHM		
	VAS	FDTD	EXP	VAS	FDTD	EXP
#1	4.00	3.92	4.21	0.33λ	0.34λ	0.33λ
#2	7.00	6.90	6.80	0.40λ	0.40λ	0.39λ
#3	9.97	9.76	10.12	0.42λ	0.43λ	0.47λ

**Table 3:** The experimental dispersion features of the SOLs at three distinct wavelengths.

SOL	*f* (μm)			FWHM		
λ (nm)	532	640 ^a^	730	532	640 ^a^	730
#1	5.06	4.21	3.28	0.32λ	0.33λ	0.41λ
#2	9.28	6.80	5.46	0.34λ	0.39λ	0.49λ
#3	12.50	10.12	7.80	0.40λ	0.47λ	0.55λ

^a^ Denotes the designed wavelength of SOLs.

**Table 4:** The simulated and experimental FWHMs and focal lengths as the radius decreases.

SOL	*f* (μm)		FWHM	
	FDTD	EXP	FDTD	EXP
*R* = 10 μm	6.90	6.80	0.38λ	0.39λ
*R* = 8 μm	6.88	7.34	0.44λ	0.51λ
*R* = 6 μm	6.72	6.54	0.55λ	0.56λ
